# Stem Cell Banking for Regenerative and Personalized Medicine

**DOI:** 10.3390/biomedicines2010050

**Published:** 2014-02-26

**Authors:** David T. Harris

**Affiliations:** Department of Immunobiology, University of Arizona, PO Box 245221, Tucson, AZ 85724, USA; E-Mail: davidh@email.arizona.edu; Tel.: +1-520-626-5127; Fax: +1-520-626-2100

**Keywords:** stem cells, banking, cord blood, MSC, cord tissue

## Abstract

Regenerative medicine, tissue engineering and gene therapy offer the opportunity to treat and cure many of today’s intractable afflictions. These approaches to personalized medicine often utilize stem cells to accomplish these goals. However, stem cells can be negatively affected by donor variables such as age and health status at the time of collection, compromising their efficacy. Stem cell banking offers the opportunity to cryogenically preserve stem cells at their most potent state for later use in these applications. Practical stem cell sources include bone marrow, umbilical cord blood and tissue, and adipose tissue. Each of these sources contains stem cells that can be obtained from most individuals, without too much difficulty and in an economical fashion. This review will discuss the advantages and disadvantages of each stem cell source, factors to be considered when contemplating banking each stem cell source, the methodology required to bank each stem cell source, and finally, current and future clinical uses of each stem cell source.

## 1. Background Introduction

Political and ethical controversy surrounds the use of embryonic stem (ES) cells, and significant biological and regulatory concerns limit their clinical use (the latter concern also applies to induced pluripotent stem cells (iPSC)). Aside from the moral and political controversies, ES cells are by their derivation allogeneic in nature and subject to immune rejection if used *in vivo*. Although ES cells may of themselves lack HLA expression, the mature tissues that arise from these cells do express HLA antigens and are subject to immune surveillance. Furthermore, ES cells are prone to give rise to teratoma formation when placed *in vivo*, making their clinical use problematic if not impossible. Although iPS cell generation avoids the allogenicity issue, the teratoma formation problem is still present. Investigators have tried to avoid this issue by deriving mature tissues from iPS cells for clinical use. However, this process is time consuming (4–6 months) and expensive (estimated to be $50,000 or more to derive a single cell line). It should be noted that having tissues that escape immune surveillance is not desirable, as viral infections could go unchecked leading to viremia and serious consequences. Therefore, in most instances regenerative medicine, tissue engineering, gene therapy and most forms of personalized medicine are best served by using autologous sources of stem and progenitor cells. However, the preferred sources must be easily accessible, contain large numbers of stem cells, and economical to utilize. It is our belief that there are only four such sources available: cord blood (CB; generally 30–120 cc can be collected) and tissue (CT; generally 4–10 inches are available), bone marrow (BM; generally 1000–1500 cc is available by surgical harvest) and adipose tissue (AT; generally 100–3000 cc available using either local or general anesthesia). CB and CT contain hematopoietic stem cells (HSC) (CB only) and mesenchymal stem cells (MSC) (CT primarily) that are available only at the time of birth. BM contains both HSC and MSC although not at high concentrations and these cells may be subject to the detrimental effects of age and health status. AT contains the highest concentration of MSC in the body and is easily accessible (without surgery) in small or large volumes. Of all cellular sources available for use, those containing MSC may be the most useful for clinical applications.

MSCs offer a multitude of potential applications in regenerative medicine, being able to proliferate and differentiate *in vitro* into multiple lineages [1,2,3]. Low immunoreactivity and high immunosuppressive properties make MSCs a suitable stem cell source for therapy [4,5]. It has been shown in numerous model systems that MSCs can be used to successfully treat cardiovascular [6,7], neurological [8] and musculoskeletal disorders [9] either by differentiation into competent cardiomyocytes, neuron-like cells and chondrocytes, respectively; or through a paracrine effect via the secretion of growth, anti-apoptotic and anti-inflammatory factors. In addition, various clinical trials are now underway to assess the effects of these stem cells in patients (see: http://www.clinicaltrials.gov). To date, bone marrow is the best characterized source of MSCs and most clinical data has been based on bone marrow studies. However, there are limitations to the use of bone marrow-derived MSCs (BM-MSCs); e.g., a painful acquisition process, use of extensive anesthesia during the harvest, and low cell yield per cc of tissue. Further, BM-MSCs have been shown to exhibit a decline in MSC numbers, proliferation, angiogenic and wound healing properties, and differentiation, along with enhanced apoptotic and senescent traits with advancing donor age [7,10,11]. Recently, other MSC sources have gained clinical interest for use in regenerative medicine; and adipose tissue (AT) represents one of these sources. AT-MSCs possess morphological, phenotypic and functional characteristics similar to BM-MSC [12], are stable over long term culture, expand efficiently *in vitro* and possess multi-lineage differentiation potential [3,13]. Human adipose tissue may represent a more practical autologous source of MSCs for various tissue engineering strategies. However, the effectiveness of these cells when obtained from any of these sources, and utilized in elderly patients, must be considered when contemplating cell-based therapies (see below).

## 2. Practical Stem Cell Sources

**Figure 1:**
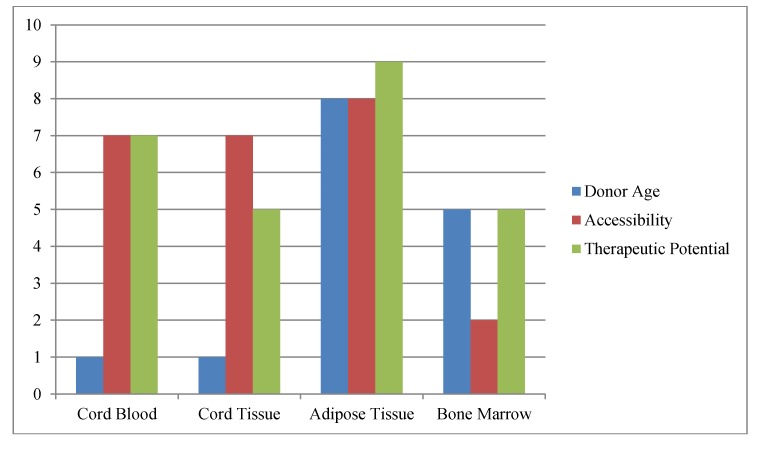
Schematic of Factors Impacting Stem Cell Banking.The most commonly available and practical sources of stem cells are compared *versus* one another for age at acquisition, ease of harvest (accessibility) and therapeutic potential. Donor age refers to total decades of life when one could acquire the stem cell sample; accessibility refers to how easy (10) or difficult (1) the stem cell sample is to collect; while therapeutic potential is used to indicate potential number of uses with (10) representing the greatest potential uses.

Stem cells can be found throughout the body, being present in many tissues and organs (e.g., heart, brain and muscle). In addition, stem cells can be isolated from the heretofore waste products of birth (CB and CT) as well as being created in the laboratory (*i.e.*, ES and iPS cells). When considering the use of stem cells for regenerative medicine and tissue engineering, one must consider the practical aspects of the endeavor (Figure 1). That is, initially and for some time to come, such therapy will not be reimbursed by insurance and must be funded by either the investigator, industry and/or the patients themselves. In addition, in order to be considered for reimbursement any new stem cell based therapy must be as efficacious as standard therapy and cannot be any more expensive. Thus, when considering a source of stem cells for use in these therapies one must identify a source of autologous tissue (to avoid immune rejection issues) that can be readily and inexpensively accessed, which contains large numbers of stem cells (not requiring expansion before clinical use). It is our belief that these constraints limit our choices of stem cell sources to bone marrow, cord blood and tissue, or adipose tissue. Bone marrow, albeit a source of MSC that has been used extensively, is expensive to harvest (with some risk to the donor) and does not contain large numbers of stem cells per cc of tissue. Therefore, this review will focus on banking stem cells collected from cord blood and tissue, or adipose tissue.

The blood in the umbilical cord and placenta after the birth of a child is comparable to bone marrow for use in hematopoietic stem cell transplantation and offers a number of advantages. In the past 20 years, more than 30,000 cord blood transplants have been performed worldwide [14]. Stem cell transplantation for hematological malignancies and genetic disorders however, is an uncommon occurrence. Research performed by several independent laboratories [15,16,17,18,19,20] has demonstrated that cord blood also contains a mixture of pluripotent stem cells capable of giving rise to cells derived from the endodermal, mesodermal, and ectodermal lineages. In addition, mesenchymal stem cells (MSC), although rare in cord blood, can be easily isolated from the cord tissue (CT) and preserved for later use [3], prompting the development of methods for the collection and cryopreservation of cord tissue [3]. Thus, CB and CT are a readily available stem cell source for use in tissue engineering and regenerative medicine applications, which are hypothesized to be more frequent events than the need for hematopoietic stem cell transplant. It is estimated that almost 1 in 3 individuals in the United States, or 128 million people, could benefit over their lifetime from regenerative medicine, including therapies for cardiovascular, neurological, and orthopedic diseases [21]. However, the absolute numbers of these hematopoietic stem cells (HSC) are limited and to date it has not been possible to successfully replicate HSC. However, MSC can be easily expanded *ex vivo* and CT represents a viable source of such cells. Absolute numbers of CT-MSC again are limited (estimated at 250,000 to 10 million total cells) requiring extended *in vitro* expansion prior to use; which invites additional regulatory oversight, costs and possible culture-induced senescence issues. Fortunately, recent work from our laboratory has demonstrated that CT-MSC and AT-MSC are equivalent in regenerative medicine potential [3]. Although CT-MSC proliferate to a greater extent *in vitro*, the ability to easily acquire 100–1000× more MSC numbers with AT (at almost any time in a patient’s life) makes adipose tissue the preferred source for clinical banking of MSC. Additionally, detailed FACS analyses showed no differences in phenotypic expression of CD3, CD14, CD19, CD34, CD44, CD45, CD73, CD90 and CD105 molecules between CT-MSCs and AT-MSCs. Cells from both sources efficiently differentiated into adipose, bone, cartilage and neuronal structures as determined with histochemistry, immunofluorescence and real-time reverse transcriptase polymerase chain reaction. To date, the vast majority of studies have concluded that both tissues are suitable sources of stem cells for potential use in regenerative medicine.

Generally, mesenchymal stem cells (MSCs) are thought of as being non-hematopoietic cells with multi-lineage potential that hold great promise for regenerative medicine. In the past decade, much research has been devoted to bone marrow-derived MSCs. Many clinical and pre-clinical studies have shown that these BM cells can be successfully used for the treatment of various diseases and disorders [22,23]. Studies have shown differentiation into mesenchymal and non-mesenchymal cell types, including adipocytes, osteoblasts, chondrocytes, muscles and neurons [24,25,26]. However, bone marrow-derived MSCs are not ideal because of the painful isolation process, the low cell yield and the often older adult age of the cell donor. Lower cell numbers and a reduced proliferative and differentiation capability owing to *in vitro* and *in vivo* aging [10,27] make bone marrow a less ideal source of MSCs. Use of alternative MSC sources is an important aspect to consider for regenerative medicine applications.

Human adipose tissue may provide the best alternative source of MSCs for tissue engineering and regeneration. Adipose tissue is a convenient, abundant and readily available source of stem cells; the harvest procedure is less invasive than bone marrow aspiration and is associated with little discomfort for the patient. Adipose tissue has 500-fold more stem cells than bone marrow per gram of tissue [28,29]. Thus, hundreds of millions of MSCs can be easily obtained from a single individual because large adipose samples can be obtained from multiple harvest sites. In addition, the cells proliferate rapidly *in vitro* and demonstrate low levels of senescence after months of *in vitro* culture [29,30]. Cell-based therapies need viable and ample numbers of cells for regenerative medicine. For autologous therapies, the cells should be available soon after injury, as would be the case for adipose tissue and previously banked cord tissue.

## 3. Stem Cell Banking Approaches

### 3.1. Cord Blood

Most CB banks have adopted the use of small (*i.e.*, pediatric) blood bags (approximately 250 cc in size); although collections can also be made with 60 cc syringes. Bag collections are preferred as collection of the blood (and subsequent processing) occurs in a closed system (preferable for most regulatory stipulations). However, bag collections must not be left unattended in order to prevent unintended contamination or loss of blood flow from occurring. Routinely, collections are completed within 5 min (prior to placental expulsion after clamping and sectioning of the cord) by accessing the umbilical vein. Alternatively, one can wait for delivery of the placenta and collect the blood directly from the expelled placenta. 

The vast majority of the cellular constituent in the cord blood collection is red blood cells (RBC), followed by neutrophils (making up 70%–80% of the leukocyte population). In reality only the mononuclear (MNC) fraction (20% of the leukocyte population), which contains the stem cell population, is needed for banking. The stem cells make up approximately 1% of the MNC fraction. CB has a very high hematocrit and RBC can make up more than half of the collection by volume. Thus, to facilitate the banking procedure, the vast majority of CB collections are RBC-depleted or reduced prior to cryopreservation. Several methods are in use to accomplish this goal including Hespan sedimentation to obtain a modified buffy coat [31], density gradient centrifugation (Ficoll method) to obtain enriched MNCs [32], and two automated processes (Sepax^®^ from Biosafe SA, Eysins, Switzerland and the AutoXpress Platform^®^ (AXP) from Thermogenesis, Rancho Cordova, CA; [33,34]) that result in a buffy coat product. The Hespan, Sepax, and AXP processing methods result in cord blood products containing all nucleated cell populations found in the original collection (MNC, neutrophils, some normal as well as nucleated RBC), while the Ficoll method enriches for the stem cell-containing MNC subpopulation (generally greater than 85% of the final cell composition are MNC with a few neutrophils and nucleated RBC). Total cell counts obtained in the final Ficoll product are generally 50% or less of the cell counts found in the other processes for this reason, although absolute stem cell recovery is similar. The AXP and other automated methods allow for greater sample throughput with fixed personnel numbers (increasing the economy of operations) than manual methods. The AXP device is also an FDA-cleared and functionally closed system (which is recommended under the current regulatory guidelines [35]). In addition, there are a few CB banks that perform plasma reduction as a means of volume reduction prior to banking. It is thought that there may be important components in the non-leukocyte fraction that would be important for clinical use. In addition, the RBC may always be removed later after thawing [36].

An average cord blood collection is generally 70–80 mL of blood from a typical full-term (40 weeks), live birth, containing an average of slightly more (or less) than (850–1100) × 10^6^ total nucleated cells (personal experience). The majority of CB banks currently store CB units in multiple aliquots. Generally, this task is accomplished by the use of a freezing bag divided into multiple compartments. Multiple aliquots allow for future use of the stem cells in cell expansion, gene therapy, or for regenerative medicine uses, which may only require a fraction of the frozen unit. Thus, it would not be necessary to thaw the entire unit unless absolutely needed, avoiding the damaging effects of repeated episodes of freezing/thawing. Multiple aliquots also allow for potency testing and confirmation of identify of the unit. Commercially available freezing bags now routinely provide for at least two unit aliquots comprising a 20% and an 80% fraction of the processed unit in separate compartments. Alternatively, one may store small numbers of cells (and plasma if desired) in small 2.5 cc cryovials.

### 3.2. Cord Tissue

An additional source of stem cells that can be simultaneously obtained at birth is the cord tissue (CT) itself which is a ready source of MSCs. CT can be collected and banked as a future source of stem cells for regenerative medicine and tissue engineering. In addition to MSCs, CT also contains endothelial and epithelial precursor cells that may be useful for these applications [37,38]. Theoretically, MSCs could be isolated either from cord blood or from cord tissue [37,38]. However, multiple investigators have reported significant difficulty in isolating sufficient numbers of MSC directly from cord blood (as well as from peripheral venous blood) for clinical use. An example of the difficulty in reproducibly isolating large numbers of MSCs from cord blood was reported recently by Zhang *et al*. [39]. Their work revealed the specific optimal parameters needed to be met in order to obtain significant numbers of blood-derived MSCs; including CB collections larger than 90 cc and processing of such samples within 2 h of birth. Even when these restrictions were met the absolute numbers of MSCs isolated from CB were too low for immediate use (in the hundreds to thousands of cells); although the MSCs could be rapidly expanded *in vitro* to 1 × 10^9^ MSC in several weeks to several months. Thus, in reality, CT is really the only clinically feasible source of MSCs at the time of birth [40,41,42,43]. 

CT is derived from the human umbilical cord that develops during gestation to support the development of the fetus. The average length of the cord itself is between 30 and 50 cm depending on a variety of factors including maternal age, heath and ethnicity [44]. Stem cells have been identified in various anatomical locations throughout the cord tissue including the amniotic compartment, the Wharton’s jelly and the perivascular space surrounding the blood vessels [45]. Numerous papers have described various methods to isolate the stem cells contained within the CT, few of which are relatively facile; including “scraping out” the Wharton’s jelly from cut up cord pieces, isolation and digestion of the Wharton’s jelly directly, culture of cord tissue explants, culture of amnion explants, digestion of isolated blood vessels from CT, and isolation/culture of venous epithelial stem cell tissues [46,47,48,49,50,51,52,53,54,55,56,57,58,59,60,61,62]. Regardless of the isolation methodology used, one generally finds that the predominant stem cell population obtained is the MSC [45]. However, there are also present endothelial, epithelial and hematopoietic stem cells depending on which isolation protocol is utilized. The total number of MSC that can be obtained varies widely in the literature and may be dependent again on the method used; ranging from as low as 25,000 MSC/cm of CT to as high as 5 × 10^6^ MSC/cm of CT [45,47,49,62,63,64]. If it is assumed that the average length of CT that could be readily obtained at birth was 30 cm [64] then the number of freshly isolated MSC would range from 750,000 to 150 × 10^6^ cells assuming every cell isolated was actually a MSC [65]. Thus, the limited number of MSC that can be obtained immediately upon isolation without need for *in vitro* culture expansion is a major disadvantage for clinical use of CT-MSC.

Regardless of whether it is actually possible to reproducibly obtain the numbers of MSC claimed at the high end of the CT range noted above, generally insufficient numbers of MSC would be obtained from a typical segment of CT to allow for immediate use in clinical applications (e.g., tissue engineering or regenerative medicine) without prior expansion *in vitro*. Clinical grade MSC expansion would require a minimum of Good Tissue Practice (GTP), if not Good Manufacturing Practice (GMP)-qualified facilities, an investigational approval from the FDA (in terms of an Investigational New Drug (IND) protocol or Biologic License Agreement (BLA)) and additional resources (time and monies) in order to be accomplished successfully. If one could reproducibly obtain 150 × 10^6^ MSC from a 30 cm CT one would have sufficient MSC for some but not many applications (as many applications require (10–30) × 10^6^ cells/kg body weight based on our 20 year experience as well as common cell thresholds used for most cord blood transplants). Although the desired regenerative medicine end-use will ultimately determine what cell dose is needed, many applications generally will require some period of *in vitro* MSC expansion. Thus, currently there is no ready-to-use, out of the box, clinical trial-ready CT-MSC methodology available (in contrast to other adult sources of MSC).

Different methods may be used for successful isolation of MSCs from cord tissue including enzymatic [56,66] and non-enzymatic digestion [58,59,67]. However, there have been some conflicting reports regarding the type of MSC and the “multi-lineage differentiation potential” of CT-MSCs [59,66,68,69,70]. Regardless, CT must and can be collected at the time of birth and banked frozen for extended periods of time prior to expected use. To date no study has examined the effects of cryopreservation on the utility of CT-MSCs isolated from thawed cord tissue for use in regenerative medicine and tissue engineering. Cord tissue (CT) is generally obtained from full term deliveries. All samples should be obtained with written consent from the donors. Most collections are 5–10 inch tissue pieces cut from the umbilical cord with sterilized scissors and processed within 24 h. Specifically, the CT is cleaned and sterilized with alcohol and betadine. It is then cut with sterile scissors and a 4–8 inch segment is placed into a sterile container with transport buffer. The transport buffer contains isotonic saline with 10 U/mL heparin, 1% human serum albumin (HAS), Penicillin-Streptamycin, Gentamycin, and Amphotericin. The sample may be held and transported at room temperatures for up to 48 h. Upon receipt the CT sample is placed in isotonic saline followed by a 70% ethanol wash, and a final sterile saline wash. The CT is then cut into small 5 mm ringlets or minced into small pieces using a sterile scalpel. The CT pieces are then placed in isotonic saline containing 0.1 mol/L sucrose, 20% autologous plasma and 1.5 mol/L DMSO for 30 min at 4 °C on a rocking platform. Samples are frozen in 4.5 cc cryovials (~1.0–1.5 grams total/cryovial) using a controlled rate freezer to −180 °C. Samples are stored in liquid nitrogen dewars. 

When required, CT can be thawed at room temperature for 30 s, followed by a complete thaw in a 37 °C water bath (approx. 2 min). The tissue strips may then either be washed extensively at 4 °C to remove DMSO, or a step-down procedure may be used to remove the cryoprotectant. This latter procedure involves the washing of the tissue by agitation for 5 min in each of the following buffers at 4 °C:

- 1.5 mol/L DMSO in PBS with 20% autologous plasma & 0.1 mol/L sucrose,
- 1.0 mol/L DMSO in PBS with 20% autologous plasma & 0.1 mol/L sucrose,
- 0.5 mol/L DMSO in PBS with 20% autologous plasma & 0.1 mol/L sucrose, and
- 0.0 mol/L DMSO in PBS with 20% autologous plasma & 0.1 mol/L sucrose.

Finally, a last wash in PBS containing 20% autologous plasma is used, followed by re-suspension in PBS/20% plasma for analysis, culture or clinical use. MSCs from cord tissue can then be isolated using a non-enzymatic digestion procedure as described [68,69,70]. Briefly, pieces of cord tissue are extensively washed with PBS containing penicillin and streptomycin in a 100 mm petri-plate. The tissue is minced into fine pieces that are placed into a 25 cm^2^ culture flask. After 4–6 days the pieces are removed and cultured in a new flask. In 10–14 days cell colonies can be observed. The cells from both flasks are then harvested using trypsin-EDTA and pooled. A total of 25,000 cells are then cultured in each subsequent 25 cm^2^ culture flask in alpha-MEM expansion medium.

### 3.3. Adipose Tissue

All adipose samples should be obtained with written consent from the donors and according to any other requirements of the local Institution Review Board (IRB). Adipose tissue samples are generally obtained from scheduled liposuction procedures, or by syringe harvest performed under local anesthesia. The lipoaspirates should be processed and cryopreserved within 24–36 h of collection. For cryopreservation, the tissue is washed extensively with isotonic saline, and the washed tissue slurry is directly placed in a cryo-container (generally a cryobag) and an equal volume of pre-cooled dimethylesulfoxide (DMSO) solution (70% Lactated Ringer’s buffer, 20% serum or HSA, 20% DMSO) is added slowly over several minutes at 4 °C. The cryo-container is generally mixed at 4 °C for 20–30 min to allow for cryoprotectant equilibration. Cryopreservation is performed using a controlled rate freezer to −180 °C before final submersion in liquid nitrogen for long term storage [71]. In our experience we have not seen significant differences in AT-MSC harvest by tumescent liposuction, VASER liposuction, power-assisted liposuction or various forms of laser liposuction (although we have not analyzed all possible permutations that are commercially available). In addition we have not observed any deleterious effects of anesthetic choice on the clinical utility of the harvested AT-MSC (*i.e.*, Lidocaine and Marcaine were equivalent).

When needed, frozen tissues should be thawed rapidly in a 37 °C water bath. Immediately after thawing the cryopreservation solution must be diluted with expansion medium (α-MEM) supplemented with FBS, HSA or other source of protein to avoid deleterious effects of the cryoprotectant at elevated temperatures [72]. Adipose tissue may then be utilized as is or further digested as described above. Adipose derived MSCs (AT-MSC) may be isolated by enzymatic digestion as described [12,13] if desired. The tissue slurry can be digested with 0.2% type IV collagenase by incubation at 37 °C for 15 min. MSC may be expanded by culture in medium consisting of α-MEM media supplemented with 10% FBS (or HSA) and 1% each of non-essential amino acids, sodium pyruvate, l-glutamine and streptamycin/penicillin solution. Expansion medium is also added to the digested tissue to neutralize collagenase. The infranatant is centrifuged at 150 g for 10 min to obtain cells of the stromal vascular fraction (SVF). AT-MSC are then plated in 25 cm^2^ culture flasks and maintained in a humidified atmosphere at 37 °C with 5% CO_2_. After two days of culture, non-adherent cells are removed by changing the medium to leave a homogenous MSC population. Medium is changed twice weekly thereafter. 

## 4. Common Stem Cell Uses

Cord blood stem cells have been used in the clinic to treat malignant and genetic blood disorders for more than 20 years now. Over the past decade CB has made its way into multiple clinical trials for use in regenerative medicine applications (http://www.clinicaltrials.gov and see below). It is only recently that CT stem cells have made their way into the clinic; and it is only now that clinical comparisons to bone marrow MSCs can be made. A recent report from Xue *et al*. examined the use of CT-MSCs in patients with non-healing bone fractures [71]. This study reported significant clinical benefit from intravenously infused MSC. CT-MSCs are also showing positive results in treating GVHD following hematopoietic stem cell transplantation. Two pediatric patients with severe steroid-resistant GVHD were infused with CT-MSCs. The GVHD improved dramatically in both patients following infusion of CT-MSCs, although one patient received multiple infusions of MSCs until the course of treatment was complete [73]. CT-MSCs have also been evaluated for potential therapeutic benefits in autoimmune diseases. Liang *et al*. reported that CT-MSCs stabilized the disease course of a patient with progressive multiple sclerosis that was not responsive to conventional treatment [74]. A subsequent study from the same group reported dramatic improvements in a patient with systemic lupus erythematosus following intravenous infusion of CT-MSCs [75]. Importantly none of the case reports indicated adverse effects associated with infusion of CT MSCs. Based on promising *in vitro* and *in vivo* results for a wide range of conditions, MSCs, primarily from bone marrow, are currently being investigated in more than 350 planned, ongoing, or recently-completed clinical trials for conditions including ischemic injury (heart attack, stroke, critical limb ischemia), autoimmune diseases (type I diabetes, MS, SLE), inflammatory conditions (COPD, Crohn’s disease), orthopedic applications (bone fractures, cartilage injuries, osteoarthritis, osteogenesis imperfecta), and transplantation (both stem cells and organs). Specifically, AT-MSC are currently being used in more than 100 ongoing clinical trials for many of the same disease settings.

As might be expected, when it is possible to harvest and bank MSCs from various tissues at different times throughout the lifespan of the donor, the question will always be raised as to why should someone use younger as opposed to older MSC? This topic will be discussed in more detail below. However, numerous studies have indicated that MSC isolated from older donors, as well as from patients with longstanding (chronic) disease conditions are neither as prevalent [76,77,78] nor as potent [10,79,80,81,82] as those isolated from younger and healthier donors. MSCs collected from older donors and/or donors with chronic diseases (e.g., coronary disease, COPD, *etc.*) seem to be less able to differentiate into the different cell types needed for tissue engineering [10,76,79,80,81,82], less able to proliferate and expand to achieve cell concentrations that would allow for multiple treatments [10,79,80,81,82], and are more prone to die off during culture and use [80]. Thus, younger stem cells are likely to be more useful for regenerative medicine applications than older MSCs. Finally, there is anecdotal evidence that increased MSC donor age and disease status negatively impacts clinical utility and successful clinical outcomes. Data on the success rates of treatment of patients with myocardial infarction and chronic heart disease is mixed, but seems to be negatively correlated with patient age and chronic disease [83]. 

### 4.1. Hematologic Settings: CB and CT

Cord blood is unique in that it contains hematologic stem cells and thus may be used to reconstitute the blood and immune system after chemotherapy, radiation and stem cell transplant. Although bone marrow is similar in this regard, neither cord tissue nor adipose tissue has this capability. Cord blood may be used to treat more than 80 malignant and non-malignant hematologic conditions requiring transplant. In addition, CB may also be used for regenerative medicine and tissue engineering applications, as can cord tissue and adipose tissue MSC [14,21]. In the transplant setting CT-MSCs have shown positive results in the treatment of GVHD following hematopoietic stem cell transplantation. Wu *et al*. [73] found that CT-MSC had superior proliferative potential and increased immunosuppressive effects as compared to bone marrow MSC. Two pediatric patients with severe steroid-resistant GVHD were infused with *ex vivo* expanded CT MSCs. The GVHD improved dramatically in both patients following infusion of CT-MSCs, although one patient needed to receive multiple infusions of MSCs over the course of treatment [73]. CT-MSCs have also been evaluated for potential therapeutic benefits in autoimmune diseases. Liang *et al*. [74] reported that CT-MSCs stabilized the disease course of a patient with progressive multiple sclerosis that was not responsive to conventional treatment [74]. A subsequent study from the same group reported dramatic improvements in a patient with systemic lupus erythematosus following intravenous infusion of CT-MSCs [75]. Importantly none of the case reports indicated adverse effects associated with infusion of CT-MSCs. 

When contemplating which applications will most likely be first to the clinic as well as ones applicable for most people, we believe that there are three primary stem cell indications: orthopedic (e.g., cartilage repair in the articular joints), cardiovascular (e.g., heart attack), and neurological (e.g., stroke). Most individuals are likely to experience one or more of these categories of problems during their lifetimes and thus be candidates for regenerative medicine therapy. In the following discussion we will focus first on applications in which CB and CT have been used, with a separate section for AT afterwards.

### 4.2. Neurological Settings

#### 4.2.1. CB

Cerebrovascular diseases are the third leading cause of death in the United States, not including the multitudes of individuals who survive only to suffer debilitating lifelong issues. Cerebral ischemia (CI) is by far the most prevalent cause of stroke (87%, http://www.americanheart.org [83]). Approximately 700,000 people in the United States are affected by stroke annually; and 1 in 16 Americans who suffer a stroke will die from it [84]. The brain is extremely sensitive to hypoxia and some degree of tissue death is likely from stroke. At a relatively young age the brain loses most of its plasticity so any significant tissue death can be profoundly devastating. Interestingly, in young children the brain is very plastic and very large portions of the brain can be removed (such as removal of tumors or hemispherectomy for severe seizures) with relatively little long term neurological damage. These facts suggest that younger neural cells might have a greater capacity to regenerate the injured brain. 

Nowhere has the potential significance of CB stem cell therapy for the treatment of neurological disease been greater than in this area of stroke therapy. As early as 2001, it was demonstrated that the infusion of CB stem cells into rats in the commonly used MCAO (middle carotid artery occlusion) model of stroke could reverse many of the physical and behavioral deficits associated with this disease [85]. Studies demonstrated that direct injection of the stem cells into the brain was not required [86], and in fact, beneficial effects could be observed even if the stem cells did not actually home into the target organ (probably via the release of growth and repair factors triggered by the anoxia) [87,88]. The beneficial effects seemed to be dose-dependent and could reduce the size of the infarcted tissue [89]. It appeared that multiple progenitor populations in CB were capable of mediating these effects [90]. Significantly, unlike current pharmacological interventions that require treatment within the first few hours after stroke, CB stem cell therapies were effective up to 48 h after the thrombotic event [91]. In fact, administration of CB stem cells immediately after the ischemic event may be detrimental in that the inflammatory milieu may be toxic to the administered stem cells.

The majority of reported studies [90,91,92,93,94,95,96,97] have shown that CB administration in stroke models resulted in some degree of therapeutic benefit with no adverse effects. Neuroprotective effects [90,91,92,95,96,98] as well as functional/behavioral improvements [91,96,97] from CB therapies have been widely reported. Neurological improvement was accompanied by decreased inflammatory cytokines [89], by neuron rescue/reduced ischemic volume [90,91,92], as well as by lowered parenchymal levels of granulocytic/monocytic infiltration and astrocytic/microglial activation [91]. Thus, the mechanisms behind the observed beneficial effects afforded by CB therapies included reduced inflammation [92], protection of nervous tissue from apoptosis [90] and nerve fiber reorganization [90]. These observations are particularly encouraging as it implies that CB therapy can mediate both direct restorative effects to the brain as well as tropic neuroprotection. Many of the published studies lend support to this trophic role, in that several investigators reported [89,90,97] neural protection with little to no detection of CB cells engrafted in the brain. The level of engraftment in the brain appeared to be a function of the route of CB administration. When CB was administered intravenously [89,97,98,99], little or no CB migration to the brain was found. However, when CB was given intraperitoneally [99] there was evidence of neural restorative effects. Early studies have also shown benefit in animal models of hemorrhagic (as opposed to embolic) stroke [96]. For additional information one is referred to the recent review on cell therapies for stroke found in reference [100]. 

In addition to stroke, CB stem cells have been used in other nervous system injury models, two of which have now instigated clinical trials. Lu *et al*. [101] demonstrated that intravenous administration of CB mononuclear cells could be used to treat traumatic brain injury in a rat model. In this model the CB cells were observed to enter the brain, selectively migrate to the damaged region of the brain, express neural markers, and reduce neurological damage. Similarly, CB stem cell transplant could also alleviate symptoms of newborn cerebral palsy in a rat model, with improved neurological effects [93]. These observations have now been turned into clinical therapies (see below). Early, albeit anecdotal, reports have indicated beneficial effects from the CB mononuclear cell infusions [102]. Several investigators have begun planning clinical trials to treat children with hypoxic/ischemic and traumatic brain injury utilizing autologous cord blood stem cell infusions 

The observation that CB stem cells can become different types of nervous cells extends its utility to other areas of neurological damage, including spinal cord injury. Spinal cord injured rats infused with CB stem cells showed significant improvements five days post-treatment compared to untreated animals. The CB stem cells were observed at the site of injury but not at uninjured regions of the spinal cord [85]. This finding is supported by another study demonstrating that CB stem cells transplanted into spinal cord injured animals differentiated into various neural cells, improved axonal regeneration and motor function [103]. Significantly, in a recently reported clinical use of CB stem cells to treat a patient with a spinal cord injury [104] it was stated that transplantation of CB cells improved her sensory perception and mobility in the hip and thigh regions. Both CT and MRI studies revealed regeneration of the spinal cord at the injury site. Since the CB stem cells were allogeneic in origin it will be significant to determine if immune rejection or other immune-mediated problems occur that might jeopardize the early improvement. Neither additional patients nor additional studies in this area have been reported. However, the use of CB stem cells for spinal cord injury seems to be the next logical clinical trial. Large numbers of children are unfortunate enough to suffer a spinal cord injury at an early age (e.g., diving into a pool, car accidents, falls, *etc.*) and it would be expected that a significant number would have autologous cord blood banked and available for treatment.

#### 4.2.2. CT and AT

CT-MSC can be differentiated into neuron-like cells *in vitro* [3] which may indicate that there could be applications for neurological conditions like stroke, Parkinson’s disease and Alzheimer’s disease. Animal work in ischemic stroke has also shown promising results [105,106,107,108]. In addition CT-MSCs have shown promising results in intracerebral hemorrhage models [109] and in the treatment of spinal cord injuries [110,111,112,113]. Finally, the anti-apoptotic effects of MSC have provided beneficial effects in animal models of Parkinson’s disease [47,114].

AT-MSC can also be differentiated into neural tissue [3] which has led to its introduction into several human clinical trials. Due to a longer history of clinical work with BM-MSC and its similarity, AT-MSC are currently being investigated as a therapeutic approach to spinal cord injury [111], stroke [109,115,116], and Parkinson’s disease [47]. 

### 4.3. Cardiovascular Settings

#### 4.3.1. CB

Cardiovascular disease is the leading cause of morbidity and mortality for both men and women in the United States. Approximately one million people die of cardiovascular disease annually despite medical intervention. Coronary artery disease comprises approximately half of these deaths. As heart cells have a limited capacity to regenerate after myocardial infarction (MI), application of exogenous stem cells seems a logical alternative for therapy. Recently, numerous pre-clinical and clinical studies examined the use of adult hematopoietic stem cell sources (see ref. [84] for details and additional references). To date, only non-embryonic stem cells have been examined in clinical trials due to political, ethical and biological constraints. There have been no clinical trials using CB stem cells for cardiovascular disease. The lack of clinical trials has been due to the relative youth of the CB banking industry. However, although no clinical trials utilizing CB stem cells for heart failure have been conducted to date, a number of pre-clinical animal studies have been performed [116,117,118,119,120,121,122]. Several common observations were noted in these studies regardless of the protocols utilized including selective migration of the CB stem cells to the injured cardiac tissue, increased capillary density at the site of injury, decreased infarct size, improved heart function and a general lack of myogenesis. These observations are thought to be due to the production of angiogenic factors and induction of angiogenesis/vasculogenesis [6,116,123]. In fact, work done by Gaballa *et al*. [84,116] in myocardial infracted rats showed that CD34+ CB stem cells induced blood vessel formation, reduced infarct size and restored heart function. The effects were thought to be due to the release of angiogenic and growth factors (e.g., VEGF, EGF and Angiopoietin-1, 2) induced by hypoxia as shown by gene array analyses. This work demonstrated that cord blood stem cells could be induced to become/differentiate into endothelial-like cells. Interestingly, as a prelude to human clinical trials for MI, it has been shown that it is possible to isolate therapeutic cells from CB using a clinical grade apparatus making the transition from bench to bedside a bit more facile. Finally, work from numerous groups seems to indicate that more than one population of pluripotent cells contained in CB is capable of mediating this effect as shown by the ability of CD34+, CD133+ and CD45− cells to induce cardiac repair after MI [116,117,121,122,124]. Even more important, the numbers and potency of these cells found in CB seem sufficient for adult human applications as shown by work performed in a porcine model [121].

Aside from its application to MI, CB stem cells via the exertion of its angiogenic capability also appear to be useful for the treatment of various ischemic diseases. Many investigators have demonstrated that not only does CB contain cells displaying the phenotypic characteristics of endothelial precursors that are responsible for blood vessel formation, but that these cells are capable of differentiating into endothelial cells and becoming blood vessels [125,126,127,128,129,130,131,132]. These bioengineered blood vessels appeared similar to native blood vessels in terms of their (three layered) tissue organization as well as expression of matrix components [125,127,128,130]. Furthermore, when placed in animal models CB stem cells were able to significantly reverse the effects of ischemia in several model systems [126,129,132]. In models of hind limb ischemia, transplantation of CB stem cells or endothelial cells derived from CB stem cells appeared able to reverse surgery-induced ischemia resulting in limb salvage [133,134,135,136]. 

#### 4.3.2. AT

The use of MSC in cardiovascular disease therapy has a long history via the use of BM-MSC [84], with mixed results. Due to the phenotypic and functional similarity of BM-MSC and AT-MSC [3], a number of clinical trials using AT-MSC in this arena have been initiated (http://www.clinicaltrials.gov), including Myocardial infarction, Congestive heart failure, Stroke, Critical limb ischemia/peripheral artery disease, and Coronary ischemia. It is still too early to determine how effective this approach will be, and for which indication it will be most efficacious.

### 4.4. Orthopedic Applications

#### 4.4.1. CB

The potential of CB stem cells to generate bone and cartilage has been recently examined. It is estimated that more than one million individuals in the USA annually suffer from articular joint injuries involving cartilage, ligaments and/or tendons, as well as difficult to heal bone fractures (see http://www.arthritis.org). CB contains both ES-like and mesenchymal stem cells (MSC) capable of differentiating into both bone and cartilage [137]. In fact, when CB stem cells were placed into animals with fractured femurs there was significant bone healing. Work from the laboratories of Szivek *et al*. [138] and Harris (unpublished observations) have also examined the ability of cord blood stem cells to become cartilage in comparison to tissues derived from bone marrow MSC and adipose stem cells, with early encouraging results. 

#### 4.4.2. CT

CT-MSC have made their way into the clinic to treat non-hematological conditions. The potential of CT stem cells to generate bone and cartilage has been recently examined. It is estimated that more than one million individuals in the USA annually suffer from articular joint injuries involving cartilage, ligaments and/or tendons, as well as difficult to heal bone fractures [139]. CT contains mesenchymal stem cells (MSC) capable of differentiating into both bone and cartilage [140]. In fact, when CB stem cells were placed into animals with fractured femurs there was significant bone healing. A recent report from Xue *et al*. examined the use of CT MSCs in patients with non-healing bone fractures [71]. This study reported significant clinical benefit from intravenously infused MSC. 

#### 4.4.3. AT

AT-MSC have advanced the most in terms of clinical trials over the past several year. Currently there are human clinical trials using AT-MSC for cartilage replacement, osteonecrosis, osteogenesis imperfect, periodontitis, non-healing bone fractures, to treat benign bone neoplasms, and repair of degenerative spinal discs. Although very promising results have been obtained it is still too early to form definitive conclusions.

## 5. Factors Impacting Stem Cell Banking and Use

### 5.1. Stem Cell Origin and Age

Multiple factors may impact stem cell use in the clinic including origin and age of the donor stem cells, donor disease status, time from injury to treatment (the “treatment window”), and regulatory guidelines and restrictions. The first factor to consider is the stem cell source. That is, will stem cells be of autologous or allogeneic origin? Stem cells of autologous origin are much easier to implement from a regulatory standpoint (see below) and lessen the concern of disease transmission. In addition, autologous stem cells remove the concern of immune rejection which may be particularly important in instances where multiple stem cell injections are required [141,142,143,144]. However, a number of clinical trials have been attempted using allogeneic stem cells. Even in those trials that have been successful one can only treat patients a limited number of times before they become immunized and rejection occurs. The cost of this “off-the-shelf” approach to regenerative medicine is expensive, and generally limited to academic centers where frozen (*i.e.*, banked) allogeneic stem cells can be stored until ready for use. Finally, in instances where the injected stem cells differentiate into long-lasting tissues (e.g., neurons) the use of autologous stem cells is a must. Otherwise, rejection of the newly acquired tissue will eventually occur.

The average human life expectancy has significantly increased due to advances in medical research and improvements in general life style. Unfortunately however, human aging is associated with many clinical disorders and an inability of the body to maintain tissue turnover and homeostasis. As a result the number of elderly medical patients have also significantly increased, making them a major target population that could potentially benefit from cell based therapies. As autologous cell sources are preferred for economical and logistical reasons, the effect of donor age on regenerative potential should be determined before clinical use. In recent years, many studies have demonstrated the clinical potential of mesenchymal stem cells (MSCs), both *in vivo* and *in vitro* [7,145]. However, using MSC collected from the elderly who are most likely to benefit from this technology raises some concerns.

However, before widespread clinical use it is important to determine the potential effect of donor age on the expansion and differentiation capabilities of these cells. There is a logical assumption that organismal aging is linked to diminished organ repair due to reduced functional capacity of tissue resident stem cells. It is believed that such cells residing in the elderly are subjected to age-related changes and thus contribute less to tissue rejuvenation. Similarly, age-related diseases such as diabetes and heart failure also negatively impact the function of endogenous progenitor cells [146]. As stem cells are the basis of tissue regeneration therapies, a diminished functionality of these cells in the elderly may result in reduced efficacy of autologous cell therapies. With an increase in the aging population, cellular therapies are becoming more relevant for aged patients who are the main target population for such therapies.

Analyses have indicated that the overall yield of total nucleated and stem cells were significantly and negatively affected by donor age. Similar observations have been reported in literature by assessing the yield of bone marrow-derived MSCs and circulating endothelial progenitor cells [77,147]. These results indicated that age-related changes in MSC number should be taken into account whenever these cells are considered for clinical applications in the elderly. Although AT-MSCs from all age groups had the ability to form colonies (an indication of cell function), AT-MSC from younger donors produced more colonies containing larger numbers of cells. Other investigators have reported that the number of cells forming colonies decreased significantly with increasing donor age and is in accordance with the results of our current study [148].

However, recent studies have raised questions about the usefulness of AT-MSCs collected from aged donors [148,149]. Khan *et al*. [150] found age-related differences in osteogenic potential of AT-MSCs. These inconsistent results may be due to the different age ranges and the health status of the donors that were studied. Overall, the majority of reports found results similar to our current study; describing an overall decline in osteogenic potential with donor age (regardless of species). Murphy *et al*. [81] has also reported an age-related decline in chondrogenic potential of MSC similar to the results of our study. In combination, these findings and our osteogenic results indicate that donor age may negatively impact the use of AT-MSC for orthopedic applications which are not uncommon as one grows older. 

Numerous studies have indicated that MSC isolated from older donors, as well as from patients with long-standing (chronic) disease conditions are neither as prevalent [76,77,78,79] nor as potent [10,79,80,81,82] as those isolated from younger and healthier donors. MSCs collected from older donors and/or donors with chronic diseases (e.g., coronary disease, COPD, *etc.*) seem to be less able to differentiate into the different cell types needed for tissue engineering [10,76,79,82], less able to proliferate and expand to achieve cell concentrations that would allow for multiple treatments [10,79,80,81,82], and are more prone to die during culture and use [80]. Thus, younger MSC are likely to be more useful for regenerative medicine applications than older MSCs. This hypothesis remains to be proven, however. Finally, there is anecdotal evidence that increased MSC donor age and disease status negatively impact clinical utility and successful clinical outcomes. Data on the success rates of treatment of patients with myocardial infarction and chronic heart disease is mixed, but seems to be negatively correlated with patient age and chronic disease [85]. Ultimately, disease status of the MSC donor may prove to be more important than absolute age of the MSC donor. 

Finally, there is also some preliminary evidence that age of the stem cell recipient may also impact clinical efficacy [151]. That is, older recipients may require earlier intervention or additional therapies to achieve the same level of clinical success observed with younger recipients, regardless of stem cell donor age. Importantly, limited clinical benefit has been observed when using older stem cells in older recipients, implying that access to banked, younger stem cells may be critical to serving those most in need of regenerative therapies, the elderly.

## 6. Optimal Treatment Windows

Another significant variable impacting clinical outcome is time to therapy. It seems unrealistic to expect to treat almost any condition at almost any time just because one is using stem cells. In many instances, injury will be followed by inflammation and later by fibrosis, scarring and cell death. Inflammation at the beginning of the injury is toxic to cells and could kill the stem cells if administered too early after injury. Once fibrosis and scarring have been established the injured site is essentially “walled off” from therapy as infused stem cells will no longer have access to the damaged tissues. Thus, when is the optimal time to implement stem cell therapy? Most likely it will depend upon the type of injury and the age of the patient. Young patients will be better recipients especially for neurological injuries (as the pediatric brain is still growing until about age 7 years) as their systems are more resilient and less likely to have been damaged by long term inflammatory processes. In fact, the older the patient the more restricted the options will likely be and the more likely the patient will need to be treated sooner than later. Based on published reports as well as our own experience we would estimate that the window of opportunity for treatment of most conditions will be days to months, and most likely will require a waiting period of 48–72 h before infusion to avoid the effects of inflammation. Treatment during the optimal window of time takes advantage of endogenous repair mechanisms that employ resident stem and progenitor cells, rather than needing to construct new tissues *ex vivo* or depend on *in vivo* stem cell differentiation. Thus, prior to tissue death or necrosis but after inflammation has subsided, would seem to be the optimal treatment window. This relatively long treatment window allows an individual that has previously banked young and healthy stem cells to easily retrieve them for use in that time frame from almost any place in the world (and to be sent to almost any place in the world). 

## 7. Regulatory Oversight

Despite constant complaints regarding federal regulatory oversight of stem cell banking and clinical use, regulation is necessary to insure standardization and protection from charlatans. Cord blood banking began at the beginning of the stem cell regulatory movement and has been overseen by scientific, state and federal authorities. CB is unique in that the end-user of the stem cells (transplant physicians) is already regulated by insurers, CMS and state medical boards. Thus, regulation of the stem cell providers (*i.e.*, the banks) was easy and straightforward. Regenerative medicine and tissue engineering however, are problematic in that many physician specialties may be involved in therapy. In some instances these doctors will be at university and other academic institutions where studies will be conducted under the auspices of the local IRB (possibly along with filing a federal IND). However, in many if not most instances the treatments will be conducted at clinics and doctor offices where the basic rules of good tissue practice (GTP) are neither followed nor understood (e.g., disease transmission prevention, sample sterility, stem cell potency, and donor/recipient identity). Many of these therapies will escape federal detection until such time that patient advertising makes them aware, until someone complains, or until a patient is injured or dies. Thus, the FDA is faced with a dilemma. Patients are clamoring for therapies, untrained doctors are often ready to offer such treatments, and many stem cell providers are out for a quick profit. The FDA should (in conjunction with the AATB) regulate the collection, processing and banking of adipose tissue (AT)-derived and cord tissue (CT)-derived stem cells much as it does CB. In addition a plan must be put in place to oversee the rise of stem cell clinics to protect both patients and the stem cell industry, as well as to capture any useful data that may be derived from such “one-off” therapies. The regulatory plan should not be too onerous or expensive, and may consist of nothing more than registration and reporting requirements, with occasional unannounced inspections.

One complicating factor in this area is the general misconception held by many doctors concerning IRB approvals *versus* the need for an IND application. Having served on my institutional IRB and having been involved in IND trials, I can emphatically state that although the IRB and IND complement one another they are definitely not the same thing and one cannot substitute one for the other. IRB approvals (whether institutional or private ones) require descriptions of the proposed clinical study so determinations can be made that patients are well informed, not taken advantage of, and not unnecessarily exposed to risk (*i.e.*, an ethical study will be performed), and can be considered the local approval for a protocol. An IND is a federal approval (based on the HCT/P federal law that gives the FDA the right to regulate stem and progenitor cells as well as tissues) for use of a drug or medical device in a particular application that stipulates how a study will be performed, what disease states can and cannot be treated, what data must be collected and reported, how much one can charge for the procedure, if placebos and blinding of therapy are required, and ultimately will determine if standard of care can be issued so that CMS and insurer reimbursement can occur. If the FDA states that an IND is needed for a particular therapy or use of a particular stem cell source, then IRB approval is no substitute. Unfortunately, many doctors and their medical clinics confuse one for the other and are jeopardizing the entire stem cell field through their earnest but misguided efforts.

When the FDA was given oversight of the stem cell industry they established regulations that determine whether or not an IND application is required, based on patient risk. First, if a stem cell sample is more than minimally manipulated then it needs an IND approval (*i.e.*, in order to qualify for “351 *vs.* 361” regulation standards). Minimal manipulation was defined as processes that did not alter the composition or structure of the tissue containing the stem cells. Thus, with regard to CB, if one isolates the CD34 cells it is considered a manipulated tissue. In terms of adipose tissue-derived stem cells, if one enzymatically isolates the stromal vascular fraction (SVF) it is considered a manipulated tissue. Unfortunately, no definition is yet forthcoming concerning CT although many banks are enzymatically digesting the tissue before banking, which would seem to fit the definition of a manipulated tissue. Without a working definition for CT many stem cell banks are at risk of potentially running afoul of FDA regulations. Second, according to the FDA regulations the proposed use of the tissue needs to be in an autologous setting or for 1st/2nd degree relatives in order to not require an IND. That is, the sample needs to be used in a familial setting where disease transmission is minimized. And finally, and perhaps most confusing for many, the proposed use of the stem cells needs to be homologous in nature. That is, the stem cells need to function in the therapy as they would normally be expected to function in the body. That is, CB stem cells could be used to perform stem cell transplants for cancer without an IND (as these stem cells normally make blood and immune cells), but not to treat brain trauma or stroke. In terms of CT-MSC this definition would seem to preclude ever using these stem cells without an IND as the entire purpose of the stem cells is lost upon birth of the child. For AT-MSC use of the cells in cosmetic and some reconstructive applications would be permitted but use in treating a disease such as multiple sclerosis (MS) would not. AT-MSC regulations here are a bit confusing in that AT-MSC are widespread throughout the body, opening up the definition of homologous function to various interpretations.

This last issue is the most problematic as it could possibly completely shut down the growing field of regenerative medicine. If an IND is required for almost every use, regardless of whether it is an autologous use with an un-manipulated sample, then the field will either die quickly or progress so slowly as not to be terribly useful due to the higher costs involved and the inability to treat large numbers of patients quickly. It would seem more reasonable that as long as patient health was protected and risk was minimized through meeting the other two requirements, then adequate informed consent should protect the patient from being misled. Perhaps submission of all informed consents to the FDA would satisfy this aspect and allow the field to progress. Obviously something needs to be done and done quickly. I am personally aware of dozens of trials treating hundreds (if not thousands) of patients that have escaped any scrutiny at all, where I am convinced the physicians know little or nothing concerning sterile technique, good manufacturing processes, *etc.* It is very surprising that no one has been seriously injured or died as of yet, but without some standardization and oversight, I expect this to occur at any time.

## 8. Conclusions

The ability to bank autologous stem cells for later use has the potential to be a significant linchpin in the development and implementation of regenerative and personalized medicine strategies. CB, CT-MSC and AT-MSC offer the most economical sources of stem cells for almost everyone. Cord blood banking has been available for more than 20 years, is well established and regulated, and has been involved in more than 30,000 stem cell transplants and thousands of regenerative therapies. Cord tissue banking has become available over the past 5–7 years as an adjunct to cord blood banking. Although it appears to be regulated through its association with cord blood entities, in reality it is not. Regardless, its applicability to the clinic seems to be more limited and may eventually be replaced by other MSC sources. Recently, adipose tissue banking has become available and offers one the opportunity to readily and inexpensively store almost unlimited numbers of stem cells for future use. AT-MSCs have been involved in clinical trials for more than 10 years in more than 100 FDA-approved clinical applications. This particular stem cell source may soon replace BM-MSC as the preferred stem cell source for most regenerative and personalized medicine applications.

Clinical trials using cord blood stem cells to treat malignant and non-malignant blood disorders, cerebral palsy and peripheral vascular disease among others have been ongoing for many years now [85,152]. It is only recently that efforts have focused on the isolation, characterization and utilization of MSC found in CT. In fact, CT stem cells are just now making their way into clinical trials [73,74,75,85,141], albeit to a more limited extent than AT-MSC and after extensive *in vitro* cell expansion. The requirement for cell expansion highlights one of the major disadvantages of CT-MSC; low cell yield upon isolation which requires extensive (and expensive) *ex vivo* expansion before clinical use is possible. AT-MSC however, is well-established and has been involved in more than 100 clinical trials over the past 10 years. AT-MSCs are available from almost any patient and stem cell numbers in the hundreds of millions of cells are easily harvested for immediate clinical use. MSC (from whatever source) are probably the most useful stem cells for regenerative medicine applications, but CB can also be used for stem cell transplants to treat blood (malignant and genetic) and immune disorders, which MSC in general cannot. Regenerative medicine applications will most probably be performed for orthopedic, cardiovascular and neurological applications; meaning that MSC banking will be more important over one’s lifetime than other types of stem cell banking. However, stem cells in general need to be banked while young and healthy as older stem cells and those harvested from individuals with longstanding chronic and inflammatory diseases appear to function poorly in these situations. Finally, a lack of logical and clear regulatory oversight for the entire stem cell banking (and use) field is putting patients and the entire field of regenerative medicine at risk. Something needs to be done quickly in order to allow this clinical endeavor to reach its fullest potential and serve those with the greatest and most immediate need.
